# A 63-year-old female patient with fever, myalgias and interstitial lung disease

**DOI:** 10.1007/s00392-020-01671-4

**Published:** 2020-05-20

**Authors:** Rubi Stephani Hellwege, Jörg Henes, Simon Greulich, Meinrad Gawaz

**Affiliations:** 1 Department of Cardiology and Angiology, University Hospital, University of Tuebingen, Tübingen, Germany; 2 Centre for Interdisciplinary Clinical Immunology, Rheumatology and Autoinflammatory Diseases; Department of Internal Medicine II (Hematology, Oncology, Immunology and Rheumatology), University Hospital Tuebingen, Tübingen, Germany

Sirs:

The antisynthetase syndrome (aSS) can be detected by the presence of the autoantibodies anti-Jo-1, anti-PL7 or anti-PL12. Clinical presentation comprises symptoms of interstitial lung disease (ILD), arthritis and inflammatory myopathies. Myocardial involvement is very rare but has significant clinical impact on diagnostic, treatment and patients’ outcome.

Here, we report on a 63-year-old female patient presenting at the emergency department with fever, proximal muscle weakness, joint swelling, and a weight loss of 5 kg in five months. In the last six months, the patient suffered from non-productive cough and breathlessness. Outpatient body plethysmography revealed restrictive lung pattern with reduced total lung capacity (TLC: 3.95L, 74.5% of predicted value), forced vital capacity (FVC 2.18L, 74.7% of predicted value), forced expiratory volume in the first second (FEV1: 1.77L, 71.9% of predicted value), and diffusing capacity of lung for carbon monoxide corrected by alveolar volume (DLCOc/VA 58.5% of the predicted value), suggesting ILD. Presently, with inflammatory markers increased, the patient was admitted with suspected atypical pneumonia and empirical antibiotic therapy was started.

A high-resolution computed tomography (CT) (Fig. 1) showed ILD with bilateral diffuse pneumonic infiltrations. The bronchoalveolar lavage showed no abnormalities. Lung biopsy specimen revealed normal architecture, alveolar septa were both hypercellular and thickened demonstrating fibrosis. Some macrophages, foam cells, and focal lymphocytic infiltration were found intra-alveolar; no vascular or structural abnormalities. Microbiological screening did not detect any respiratory pathogens such as Legionella pneumophila, Mycoplasma sp., Pneumococci or Mycobacterium tuberculosis.

**Fig. 1:**
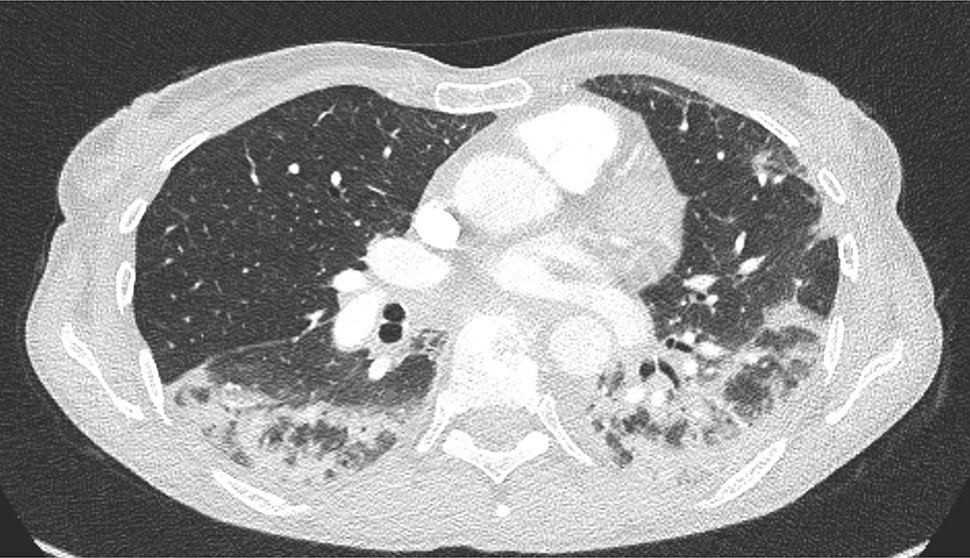
Chest computed tomography scan showing bilateral diffuse pneumonic infiltrates

Since symptoms did not improve and blood cultures were sterile, antibiotic therapy was stopped. However, laboratory tests still suggested a persistent systemic inflammation with high levels of C-reactive protein (7.6 mg/dL), creatine kinase (2961 U/L) and increased high-sensitive Troponin I (451 ng/ml) with further increase three hours later (486 ng/ml). The electrocardiogram was unremarkable; echocardiography showed a normal left ventricular function with no wall motion abnormalities. The normal size of right-sided chambers, a normal right ventricular ejection fraction, and a PAP sys of 28 mmHg made overt pulmonary hypertension unlikely. Coronary angiography demonstrated normal coronary vessels without significant atherosclerosis. Another echocardiography confirmed preserved left ventricular function but revealed a 10 mm circular pericardial effusion, suggesting perimyocarditis. Anti-inflammatory therapy with non-steroidal anti-rheumatic drugs (ibuprofen) was started but again symptoms did not improve and the pericardial effusion remained stable (9–10 mm). Moreover, the patient developed generalised oedema with bilateral pleural effusions and laboratory showed hypoalbuminemia without signs of acute liver or renal diseases. Differential diagnoses included polymyositis with cardiac involvement or systemic lupus erythematosus with polyserositis. Auto-antibody tests turned out to be positive for anti-Jo-1 (2389 U/L), anti-SSA/Ro52 (2328 U/L), anti-SMA +  + , anti-Aktin +  + , anti-Golgi-Apparatus +  + . The diagnosis of antisynthetase syndrome (aSS) was made and enhanced by magnetic resonance imaging (MRI) of the muscle and the heart (CMR) (Fig. 2a–c). Recent CMR mapping techniques allow the detection of subtle and diffuse cardiac abnormalities which might be common in rheumatic disorders depending on the disease entity. Compared to the CMR technique of late gadolinium enhancement (LGE), indicating irreversible myocardial changes (scar), increased T1-values on T1-mapping in the absence of LGE may reflect early, potentially reversible myocardial alterations [1]. Since left ventricular function continued to be preserved, endomyocardial biopsy was waived.

**Fig. 2:**
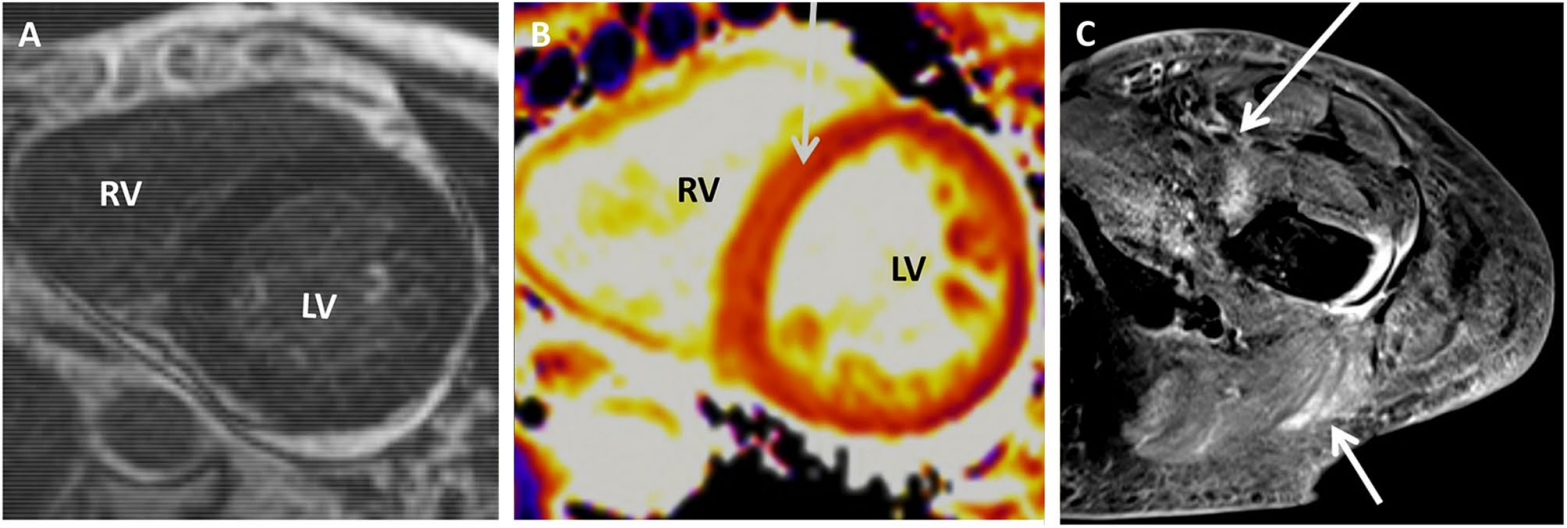
Cardiac magnetic resonance imaging (CMR) and magnetic resonance imaging (MRI) of the left thigh:. **a** CMR showed no focal late gadolinium enhancement (LGE) but recent T1-mapping technique, which is suited to detect more diffuse cardiac abnormalities, showed increased T1 levels of 1300-1400 ms (normal < 1200 ms) in the septum (arrow) consistent with an diffuse inflammatory (or fibrotic) process (**b**). *RV* right ventricle, *LV* left ventricle. **c** MRI of the left thigh demonstrating enhanced areas as a correlate for myositis (arrows showing Musculus psoas and gluteal muscle)

Corticosteroid therapy with prednisolone 1–2 mg/kg per day was initiated and the patient symptoms including muscle weakness and fevers relieved. However, creatine kinase (CK) levels remained high (> 2000 U/L). Therefore, the patient was started on azathioprine resulting in a decrease of both CK levels and pericardial effusion. Gradual clinical improvement of proximal muscle weakness and normalised CK-levels were shown at the time of her discharge. Further follow-up visits were scheduled at the rheumatological department. The patient showed a progressive relief of symptoms, reflected by the normalization of TLC, FVC, FEV1 and DLCO in the body plethysmography, regression of pulmonary alterations in the 6-month follow-up CT scan (Fig. 3), and the lack of pericardial effusion on follow-up echocardiography.

**Fig. 3.**
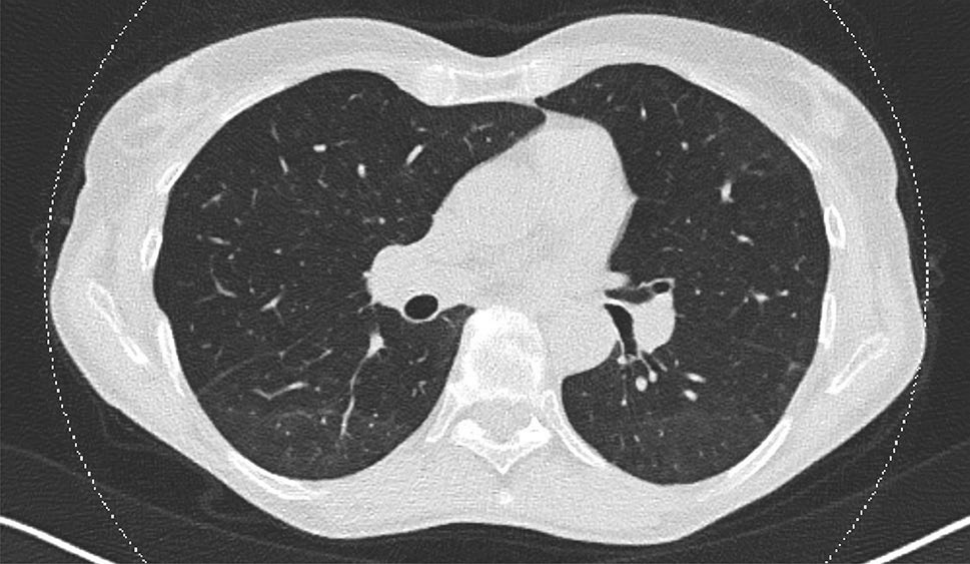
6-month follow-up chest computed tomography scan demonstrating clear regression of pulmonary alterations

The antisynthetase syndrome (aSS) is an idiopathic inflammatory autoimmune disease against the aminoacyl tRNA synthetases, enzymes that conjugated amino acids to the tRNA [2, 3]. Clinically, the aSS presents with a triad of interstitial lung disease (ILD), arthritis and inflammatory myopathies with proximal muscle weakness [2], and preponderance in females [4]. Symptoms such as fever, hyperkeratosis of the radial-fingers (mechanic’s hands) and Raynaud-phenomenon might additionally hint towards the diagnosis of aSS, although a positive myositis-specific autoantibody test result for anti-Jo-1 (histidyl tRNA synthetase), anti-PL7 (threonyl tRNA synthetase) or anti-PL12 (alanyl tRNA synthetase) are necessary to confirm the diagnosis [2]. Myositis-specific autoantibodies (MSA) target different groups of muscle proteins involving DNA repair and RNA transcription, and additional targets, such as M-i2 and Jo-1 proteins in presence of inflammation by increase of type 1 interferons [5]. Myositis associated antibodies (MAA), such as Anti-SSA/Ro may be present in patients with dermatomyositis or polymyositis [5]. In the antisynthetase syndrome, autoantibodies are against the aminoacyl-transfer tRNA synthetases (ARS), being the histidyl tRNA synthetase (anti-Jo-1) the most common target [2, 5]. Approximately, 15–20% of the patients with aSS present autoantibodies against the Jo-1 and these are associated frequently with inflammatory myopathy and cutaneous manifestations [5].

Laboratory findings in aSS include unspecific positive anti-nuclear antibodies (ANA) and elevated muscle enzymes. MRI scans often show intramuscular hyperintensities in T2-weighted MRI suggesting edema [2]. In addition, muscle biopsies showing perifascicular necrotic fibers may help to establish the diagnosis [2]. The rare finding of myocarditis (in 3.4% of aSS patients) presents a severe condition requiring immediate treatment at an intensive care unit [4]. ILD is associated with a high mortality in patients with aSS and is the most frequent complication identified in 70–89% of the patients, resulting in increased morbidity and mortality [6].

Treatment includes glucocorticoids as first-line therapy. Methotrexate or azathioprine mycophenolate mofetil, and calcineurin inhibitors, such as tacrolimus are commonly added [2, 7]. Usually, glucocorticoid therapy begins with prednisolone at a dose of 0.5–1 mg/kg daily (max. 80–100 mg per day) [2]. In severe clinical presentations, intravenous methylprednisolone at a dose of 500 mg^-1^ g daily for 3–5 days might be started, followed by an oral prednisolone dose of 0.5–1 mg/kg daily for 4–6 weeks, and then it has to be tapered [7]. Regular monitoring of muscle strength and muscle enzymes (CK) are mandatory in these patients [2, 7].

A recent case report described a patient with aSS and cardiac involvement (perimyocarditis) revealed by CMR [4]. Initially, immunosuppressive therapy with glucocorticoid and methotrexate, and an additional monoclonal therapy with rituximab showed satisfying results. However, after one month of treatment, pericarditis recurred, and could be controlled only by addition of anakinra, a recombinant human IL-1 receptor antagonist (4).

In conclusion, cardiac involvement of aSS represents a rare entity, which requires the awareness of general internists, pneumologists, rheumatologists and cardiologists to provide an interdisciplinary management, and should be consistently identified in order to establish standardized diagnostic measures and a prompt consistent therapy regimen.

## References

[1] Greulich S, Ferreira VM, Dall'Armellina E, Mahrholdt H (2015). Myocardial inflammation—are we there yet?. Curr Cardiovasc Imaging Rep.

[2] Selva-O’Callaghan A, Pinal-Fernandez I, Trallero-Araguás E, Milisenda JC, Grau-Junyent JM, Mammen AL (2018). Classification and management of adult inflammatory myopathies. Lancet Neurol.

[3] Vencovský J, Alexanderson H, Lundberg IE (2019). Idiopathic inflammatory myopathies. Rheum Dis Clin North Am.

[4] Meudec L, Jelin G, Forien M, Palazzo E, Dieudé P, Ottaviani S (2019). Antisynthetase syndrome and cardiac involvement: a rare association. Joint Bone Spine.

[5] Tartar DM, Chung L, Fiorentino DF (2018). Clinical significance of autoantibodies in dermatomyositis and systemic sclerosis. Clin Dermatol.

[6] Marie I, Josse S, Decaux O, Dominique S, Diot E, Landron C (2012). Comparison of long-term outcome between anti-Jo1- and anti-PL7/PL12 positive patients with antisynthetase syndrome. Autoimmun Rev.

[7] Cavagna L, Monti S, Caporali R, Gatto M, Iaccarino L, Doria A (2017). How I treat idiopathic patients with inflammatory myopathies in the clinical practice. Autoimmun Rev.

